# The EU response to NCDs: a content analysis on building resilient post-COVID health systems: Martina Paric

**DOI:** 10.1093/eurpub/ckac131.048

**Published:** 2022-10-25

**Authors:** M Paric, R Orhan, K Czabanowska

**Affiliations:** 1 Department of International Health, Maastricht University, Maastricht, Netherlands; 2 ASPHER, Brussels, Belgium; 3 Department of Health Policy Management, Jagiellionian University, Krakow, Poland

## Abstract

**Background:**

Non-communicable diseases (NCDs) impose a heavy burden on healthcare systems of countries in the European Union (EU). An estimated 91.3% of all deaths and 86.6% of DALYs in the EU-28 were attributable to NCDs. It is imperative that the EU act on mitigating this challenging health issue and help create trajectories for building resilient health systems. Using qualitative analysis, this study examines the question of how the European Commission 2019-2024 is planning to mitigate the impact of NCDs on health systems, while taking into account the COVID-19 pandemic.

**Methods:**

Content analysis was applied to understand how NCDs are framed and how an EU narrative is constructed to mitigate the impact of NCDs on health systems by the European Commission. A total of 44 documents were analysed, including speeches, press releases, newsletters, statements and policy documents. In vivo coding was performed using the software package ATLAS.ti 9. Unique codes were simplified and clustered into descriptive themes with a high level of abstraction.

**Results:**

This study identified five main themes: ‘health plan', ‘COVID-19', ‘future direction', ‘collaboration and solidarity', and ‘persuasion'. Themes show that the Commission is emphasising the impact of the pandemic and the relevance of policies tackling NCDs for developing EU-wide resilient health systems. By calling for more cross- and multi-sectoral collaboration, like creating a European Health Union, the Commission hopes to create the right climate for a European framework for cooperation, which can help develop harmonised and resilient EU health systems.

**Conclusions:**

Although increasing health systems resilience is high on the Commission’s agenda, it seems that there are no actionable points for Member States in terms of addressing national health policy. We recommend the Commission looks towards eliminating this observed disconnect and creating actionable points in line with Member States’ health systems’ capabilities.

**Key messages:**

• Our findings show emphasis is placed on EU-wide health policies, more robust health systems, and the collective power of the EU. However, there are no concrete actions coupled with these concepts.

• COVID-19 highlighted the limitations of national policy for protecting health systems against cross-border health threats. It tested the Commission’s resolve in pushing for more European cooperation.

